# The Multiple Dosing Effects of Platelet-Rich Plasma on Cartilage Regeneration in Knee Osteoarthritis: Randomised, Placebo-Controlled Trial

**DOI:** 10.5704/MOJ.2503.003

**Published:** 2025-03

**Authors:** A Saraf, A Hussain, V Mahipal, T Agarwal, A Kush

**Affiliations:** Department of Orthopaedics, Teerthanker Mahaveer University Medical College and Research Centre, Moradabad, India

**Keywords:** knee osteoarthritis, platelet rich plasma, VAS, WOMAC, Collagen 2-1

## Abstract

**Introduction:**

The purpose of this study was to evaluate clinical and biochemical efficacy of autologous intraarticular (IA) platelet rich plasma (PRP) compared to saline and to measure effectiveness of single and multiple doses given at monthly intervals for Kellgren-Lawrence (K-L) grade II, III knee osteoarthritis (KOA).

**Material and Methods:**

A total of 130 patients were randomised into 4 groups; PRP-1 (n=36), PRP-2 (n=34), PRP-3 (n=32) and saline (NS) (n=28), after approval from institute ethics committee (reference number: TMU/IEC/20-21/091) and was conducted in accordance with Helsinki declaration. Groups PRP-1, PRP-2, PRP-3 received single, double and triple injections of PRP whereas NS group received single saline (0.9%) injection. Assessment of outcome scores (visual analogue scale [VAS] and Western Ontario and McMaster Universities Arthritis Index [WOMAC]) was done at baseline and three, six, nine months post intervention. Serum collagen 2-1 (Coll2-1) estimation at baseline and nine months post-therapy was used for biochemical assessment.

**Results:**

Improvement in VAS and WOMAC was statistically significant and clinically meaningful (Minimal clinically important change [MCIC]; >12% of baseline and ≥2cm difference in mean for WOMAC and VAS, respectively) for groups PRP-1, PRP-2 and PRP-3 in comparison to saline (P<0.05), at every follow-up. PRP groups also exhibited a significant decrease in serum Coll2-1 at 9 months (P<0.05). On comparison among the PRP groups, multiple doses (groups PRP-2 and PRP-3) produced significantly better clinical results than single dose (group PRP-1) (P<0.05), whereas the difference in Coll2-1 levels was significant for group PRP-1 vs PRP-3 only (P<0.05).

**Conclusion:**

PRP results in clinically significant amelioration of functional and pain scores as well as significant reduction in serum levels of Coll2-1 in K-L grade II, III KOA over nine months. These benefits can be accentuated by multiple doses given one month apart.

## Introduction

Primary osteoarthritis (OA) most commonly affects the knee joint and about 85% of the global disease burden of OA is constituted by Knee osteoarthritis (KOA)^1,2^. The exact cause of OA still remains an area of research but a number of well-known risk factors are associated with it including, obesity, advanced age, female gender and genetic predisposition. The basic pathophysiology of joint damage in KOA is the disturbance of normal dynamic equilibrium between the anabolic and the catabolic mechanisms which finally lead to an irreversible matrix degeneration by matrix metalloproteinases and anti-aggregated enzymes^3,4^. This breakdown leads to a chronic inflammatory environment which is critical to the structural progression and symptom production in OA^3,4^. Chondral degeneration is the hallmark of OA but it’s being recognised as a disease of whole joint which includes the subchondral bone, synovium, and even the ligaments and menisci^4^. The clinical manifestations of KOA are pain, crepitus, stiffness, decreased joint movements, deformity and occasionally effusion, which severely impacts the quality life of an individual. Radiographic grading (grade I to IV) is done by Kellgren-Lawrence (K-L) system which is based on osteophyte formation and joint space narrowing^5^.

The goals of management in KOA are to provide a symptom free life and preserve the joint function of an individual. Conservative treatment modalities are usually the treatment of choice in early grades which include physical therapy, mobility aids, life style adjustment, activity modification, weight loss if indicated, oral pharmacotherapy in the form of acetaminophen, NSAIDS, opioids and injections of corticosteroids (CS), viscosupplements (Hyaluronic acid-HA), blood derived products given intra-articularly^3,6^. Surgical treatment options can be joint preserving methods in the form of arthroscopic lavage, shaving or debridement and osteotomies in selected patients with joint resurfacing being the treatment of choice in advanced arthritis^6^.

The focus of treatment in early KOA has now shifted towards the use of biologics which may potentially limit the joint degeneration associated with OA as other conservative methods do not have any ability to alter this degeneration. Platelet rich plasma (PRP) is a concentrated platelet sample, obtained by centrifugation of blood, has emerged as one of the most commonly used blood derived products which may serve this purpose. The release of multitude of growth factors (IGF, PDGF, VEGF, TGF-1β etc) and other active molecules (cytokines, chemokines, ECM proteins) from platelet alpha granules on degranulation directly at the injured site contribute to chondrogenesis, bone remodelling, angiogenesis, anti-inflammation and cell differentiation^7,8^. Although the chondroprotective effects of intra-articular PRP are still a matter of wide debate, its efficacy in providing symptomatic relief in short or long term (one to two years) compared to placebo, CS or HA has been reported by many level I randomised controlled trials (RCTs)^9-18^.

Despite the widespread reported clinical efficacy of PRP in KOA, various questions remain a subject of debate owing to variations in formulations, dosing, amount, quantity of platelets in the injections, type (leukocyte poor or rich) and patient characteristics (age, gender, weight). Henceforth, we conducted this study to evaluate the clinical and biochemical efficacy of autologous PRP compared to saline placebo and to understand the effectiveness of single and multiple doses given at monthly intervals in K-L grade II and III KOA.

## Materials and Methods

**Fig. 1:**
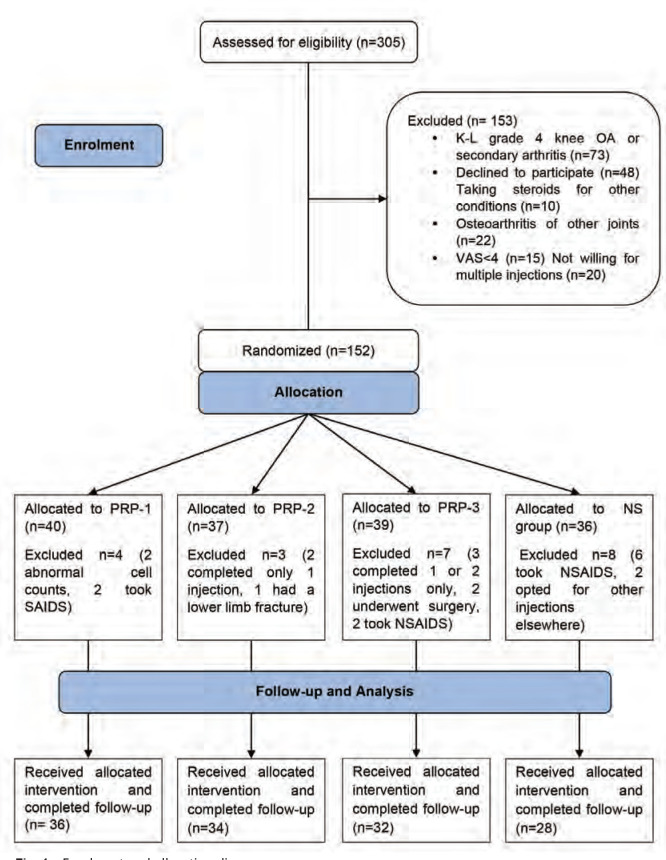
Enrolment and allocation diagram.

This prospective interventional placebo controlled injector, patient and accessor blinded study was carried out in a tertiary referral centre, from July 2021 to March 2022 (Fig. 1; enrolment and allocation diagram). Recruitment was started only after approval from the institute ethics committee (reference number: TMU/IEC/20-21/091) and it was conducted in accordance with Helsinki declaration with all patients consenting voluntarily to the intervention and follow-up.

**Fig. 2:**
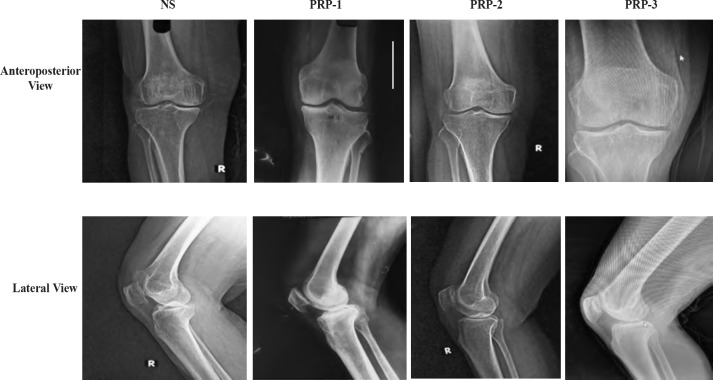
Representative knee radiographs of patients with knee osteoarthritis included in each group.

We included those patients aged 18 – 80 years presenting with clinical features of KOA, having a K-L grade II or III on standing radiographs of the knee with pain of >4cm on VAS scale over a period of one week (Fig. 2). Exclusion criteria included those with OA of multiple joint, previous knee trauma or surgery, systemic or local active infection, an uncontrolled diabetic, those who had secondary knee arthritis, including inflammatory arthritis, deformity of >10° (genu varum or valgum), coagulopathy, malignancy, prior ipsilateral knee injections within the past six months, those who were on steroids, or intake of NSAIDs within the last three weeks, haemoglobin under 11gm/dl, and platelets below 1.5x105/L.

Sampling and randomisation: For a study power of 80%, a sample size of 128 was needed (5% type I error rate, 10% drop rate), 95% confidence interval (CI), a precision of 2, with the expected population standard deviation (SD) to be 9.87 and employing t-distribution using study by Tavassoli *et al*^19^ as reference. Eligible Patients were randomised and allocated to groups with block randomisation (19 blocks with each block having 8 patients) with a computer generated program from website sealedenvelope.com. A third party handled group allocation so that the principal investigators can be blinded. The 4 groups and the treatments were as follows: group PRP-1, n=36, group PRP-2, n=34 and group PRP-3, n=32 and each group were administered with one, two and three PRP injections, respectively. Fourth group received a single injection of 3ml, 0.9% normal saline (NS group, n=28) (Fig. 1).

The syringes to be used for knee injections were covered with sterile opaque marking tape in order to keep the injector and patient blinded. The statistician doing the final assessment of outcome scores was also blinded.

PRP preparation and procedure: Under all aseptic precautions, collection of 34 – 45ml whole blood was done from antecubital vein in 8.5ml acid citrate dextrose (ACD) containers. The tubes were then centrifuged twice in a well-equipped centrifuge machine (york YSPL-1000) available in the blood bank of the institute. First centrifugation was done using a soft spin @3000rpm/minute for 3 minutes which resulted in deposition of red blood cells at the bottom, a thin layer of buffy coat or leukocytes, and the top layer of plasma containing platelets. The uppermost layer of plasma was separated from rest of the contents of the tube very carefully by a pipette so as to minimise the leucocytes and this plasma was relocated into another sterile tube which was devoid of any anticoagulant. Then this tube was given a second spin, so called a hard spin, at a higher speed @ 4000rpm/minute for 15 minutes which resulted in a platelet concentrate. The top two-thirds of this tube contained platelet-poor plasma (PPP) which was disposed off whereas as the remaining lower 3rd is PRP. A total of 3 – 5ml of PRP was obtained in this method, and 1ml was sent for leukocyte and platelet counts.

The contents in the tube were transferred to a sterile 10cc syringe with a spinal needle. All injections were conducted in a sterile environment, following all necessary precautions. Three to 4ml of either PRP or NS were administered into each knee joint through anterolateral route (just lateral to patellar tendon) without the use of local anaesthesia, with knee in 70 – 90° of flexion. Sterility was maintained all through the process of blood collection to the injection procedure.

After observing for an hour in a separate room, instructions were given orally and in written form for lifestyle modification, exercises and avoidance of NSAIDS, “chondoprotective medications” (chondroitin, glucosamine), steroids and other analgesics except tramadol, which was permitted up to a maximum dose of 50mg/day. They were provided with a “medication diary” to note down any medication intake. Groups PRP-1 and NS received single injection of PRP and NS, respectively. Group PRP-2 and PRP-3 patients received multiple injections (two and three, respectively) at interval of one month. Patients were contacted via telephone twice every month to enquire about any adverse effects or drug intake.

The primary objective was to assess the clinical efficacy of PRP by using visual analogue scale (VAS) and Western Ontario and McMaster Universities Arthritis Index (WOMAC). A 10cm line was used to mark pain intensity with zero and 10cm representing no pain and worst pain, respectively while as 4 – 6cm was considered as moderate pain. For WOMAC scores (range 0 – 96), zero and 96 represented the worst possible and best functional status, respectively. Secondary objective was to measure the pre and post intervention levels of cartilage degradation marker serum collagen 2-1 (Coll2-1) in the PRP and placebo groups. Both the primary and secondary objectives were assessed at baseline. Post interventional assessment was done at three, six, nine months of last injection, for VAS and WOMAC, while as serum Coll2-1 was estimated at final follow-up (nine months). Human Coll2-1 ELISA kits from Cusabio Technology LLC’s, Houston USA was used for estimating levels of this biomarker, which utilise the sandwich ELISA test technique.

SPSS version 21.0 for windows was used for statistical analysis. Percentages and mean±SD were used to express categorical and continuous data, whereas repeated measures analysis of variance (ANOVA) and Unpaired t-test were used for comparing mean scores between groups, and for assessment of intra/inter-group differences.

## Results

Mean age, gender (percentage), mean body mass index (BMI), K-L grade (percentage), mean VAS and mean WOMAC among the four groups were not significantly different at baseline. Majority of the patients had K-L grade III KOA (69.23%) and were female (63.07%) (Table I).

**Table I:** Baseline parameters using ANOVA, p<0.05 is significant.

Group	Intervention Groups				p-value
	PRP-1 (Single injection)	PRP-2 Double injection)	PRP-3 (Triple injection)	Normal saline (NS)	
Age (years)	52.3±7.2	56.4±6.6	58.5±6.1	55.7±5.4	0.61
Gender: Male/Female, n (%)	22/13, 62.9/37.1	24/11, 68.6/31.4	20/14, 58.8,41.2	12/16, (43/57)	0.782
KL grade 2/3, n(%)	14/21, 40/60	11/24, 31.4/68.6	8/26, 23.5/76.4	9/19 (33/67)	0.539
BMI (kg/m^2^)	25.1±3.2	25.6±4.5	26.9±4.2	26.02±4.744	0.563
WOMAC (baseline)	65.8 ±11.8	67.9±13.3	69.8±12.4	69.25±9.2	0.511
VAS (baseline)	7.7±0.9	7.1±1.3	7.5±1.3	7.464±1.3	0.863

Intra-group comparison of outcomes: The difference in baseline and post-therapy mean VAS and mean WOMAC was statistically significant for patients administered with PRP across every follow-up point (P=0.001) while as for NS group, the change was not significant (Table II).

**Table II:** WOMAC and VAS score intra-group comparison.

Gćoup	WOMAC (Unpaired t-test)			VAS (Unpaired t-test)		
	Baseline vs 3 months	Baseline vs 6 months	Baseline vs 9 months	Baseline vs 3 months	Baseline vs 6 months	Baseline vs 9 months
PRP-1	(diff 15.22, 95% CI=7.55 to 22.90, p=0.001*)	(diff 17.54, 95% CI=5.55 to 29.55, p=0.001*)	(diff 14.857, 95% CI=7.18 to 22.53, p=0.001*)	(diff 3.01, 95% CI=2.14 to 3.86, p=0.001*)	(diff 2.990, 95% CI=1.64 to 4.34, p=0.001*)	(diff 2.6, 95% CI=1.74 to 3.46, p=0.001*)
PRP-2	(diff 12.457,95% CI= 3.70 to 21.21, p=0.001*)	(diff 23.771, 95% CI=15.02 to 32.53, p=0.001*)	(diff 27.514, 95% CI=18.76 to 36.27, p=0.001*)	(diff 1.429, 95% CI=0.43 to 2.43, p=0.001*)	(diff 3.429, 95% CI=2.43 to 4.43, p=0.001*)	(diff 3.657, 95% CI=2.65 to 4.66, p=0.001*)
PRP-3	(diff 20.118, 95% CI=11.65 to 28.58, p=0.001*)	(diff 26.416, 95% CI=17.42 to 35.41, p=0.001*)	(diff 32.653, 95% CI=24.56 to 40.75, p=0.001*	(diff 3.235, 95% CI=2.23 to 4.24, p=0.001*)	(diff 4.159, 95% CI=3.10 to 5.22, p=0.001*)	(diff 4.334, 95% CI=3.38 to 5.29, p=0.001*)
NS	(diff 3.286, 95% CI= -1.678 to 8.250, p=0.1901)	diff 1.821, 95% (CI= -3.123 to 6.766, p=0.4634)	(diff -2.179, 95% CI=-9.022 to 4.655, p=0.526)	(diff -.07143, 95% CI= -0.7068 to 0.5639,p=0.8225)	(diff -0.3929, 95% CI= -0.9870 to 0.2013, p=0.1905)	(diff -.5357, 95% CI= -1.141 to 0.0698, p=0.08)

Notes – Diff: difference, CI: Confidence Interval, *: Statistically significant

Baseline Coll2-1 levels were 1521.2±409.3 pg/ml in PRP-1 group, 1481.6±348.8 pg/ml in PRP-2 group, 1394.7±478.6 pg/ml in PRP-3 group and 1452.5±449.3 pg/ml in NS group. Serum Coll2-1 levels decreased to 1200.9±293.6 pg/ml (1.3-fold), 1009.4±387.1 pg/ml (1.5-fold), and 934.7±299.6 pg/ml (1.5-fold) for groups PRP-1, PRP-2 and PRP-3, respectively at 9 months (P=0.001). No significant change was seen in NS group (1402.8±398.7 pg/ml,1.0-fold, P=0.66).

Intergroup comparison of VAS: Compared to NS, mean VAS significantly decreased for all PRP groups across every follow-up point (P=0.001). At three months, in comparison to those receiving a single PRP injection, mean VAS decreased significantly for those receiving two (diff 1.943, 95% CI=1.18 to 2.71, P=0.001) and three (diff 2.140, 95% CI=1.18 to 2.91, P=0.001) PRP injections. Similar results were seen at 6-month ([PRP-1 group vs PRP-2 group; diff 1.8, 95% CI=1.02 to 2.58, P=0.001], [PRP-1 group vs PRP-3 group; diff 1.782, 95% CI=1.0 to 2.57, P=0.001]) and 9-months ([PRP-1 group vs PRP-2 group; diff 1.971, 95% CI=1.06 to 2.88, P=0.001] and [PRP-1 group vs PRP-3 group; diff 1.931, 95% CI=1.01 to 2.85, P=0.001]). The difference in VAS scores between groups PRP-2 and PRP-3 did not reach statistical significant levels at any time point (P=0.815, P=0.998 and P=0.994 at 3, 6 and 9 months, respectively) (Table III).

**Table III:** Intergroup comparison of VAS score at various follow-up.

Duration of follow-up	Group comparison (Unpaired t-Test)	95% Confidence Interval	p-value
3 Months	PRP-1 vs PRP-2 PRP-1 vs PRP-3 PRP-2 vs PRP-3	(diff 1.943,95% CI= 1.18 to 2.71) (diff 2.140,95% CI= 1.18 to 2.91) (diff 0.197,95% CI= -0.57 to 0.97)	0.001 0.001 0.815
6 Months	PRP-1 vs PRP-2 PRP-1 vs PRP-3 PRP-2 vs PRP-3	(diff 1.8,95% CI= 1.02 to 2.58) (diff -1.782,95% CI= 1.0 to 2.57) (diff -0.18,95% CI= -0.8 to 0.77)	0.001 0.001 0.998
9 Months	PRP-1 vs PRP-2 PRP-1 vs PRP-3 PRP-2 vs PRP-3	(diff 1.971,95% CI= 1.06 to 2.88) (diff 1.931,95% CI= 1.01 to 2.851) (diff -0.40,95% CI= -0.96 to 0.88)	0.001 0.001 0.994

Intergroup comparison of WOMAC: The mean WOMAC was significantly better for PRP groups in comparison to NS group across every follow-up point (P=0.001). The difference in WOMAC was significant for groups PRP-1 vs PRP-2 at 3rd month (diff 7.34, 95% CI=0.57 to 14.11, P=0.03), 6th month (diff 8.6, 95% CI=1.32 to 15.88, P=0.016), and at 9th month (diff 11.8, 95% CI=4.44 to 9.16, P=0.001). Similarly, it was significant for groups PRP-1 vs PRP-3 as well, at 3 months (diff 9.25, 95% CI=2.43 to 16.07, P=0.005), 6th month (diff 9.85, 95% CI=2.52 to 17.18, P=0.005), and 9 months (diff 11.91, 95% CI=4.50 to 19.33, P=0.001). The difference in WOMAC scores for groups PRP-2 vs PRP-3 did not reach statistical significance at any time point (P= 0.78, P=0.91 and P=0.99 at 3, 6 and 9 months, respectively) (Table IV).

**Table IV:** Intergroup analysis of WOMAC score at various follow-up.

Duration of follow-up	Group comparison (Unpaired t-Test)	95% Confidence Interval	p-value
3 Months	PRP-1 vs PRP-2 PRP-1 vs PRP-3 PRP-2 vs PRP-3	(diff 7.343,95% CI= 0.57 to 14.11) (diff 9.252,95% CI= 2.43 to 16.07) (diff 1.909,95% CI= -4.91 to 8.73)	0.03 0.005 0.784
6 Months	PRP-1 vs PRP-2 PRP-1 vs PRP-3 PRP-2 vs PRP-3	(diff 8.6,95% CI= 1.32 to 15.88) (diff 9.852,95% CI= 2.52 to 17.18) (diff 1.252,95% CI=-6.08 to 8.58)	0.016 0.005 0.913
9 Months	PRP-1 vs PRP-2 PRP-1 vs PRP-3 PRP-2 vs PRP-3	(diff 11.8,95% CI= 4.44 to 19.16) (diff 11.914,95% CI= 4.50 to 19.33) (diff 0.114,95% CI= -7.30 to 7.53)	0.001 0.001 0.999

Serum Coll2-1: In comparison to placebo (saline), Coll2-1 levels were significantly lower for all three PRP groups at 9 months (P<0.05) but among the /PRP groups, the difference was significant for groups PRP-1 vs PRP-3 only (P<0.05).

The mean platelet count was 2.35±0.56 x105/μl (range 1.523.18 x105/ μl) and mean leukocyte count was 9.34±2.71 x 103/μl (range 5.1-14.2 x103/ μl) in whole blood. Mean platelet count was 8.36±2.82 x105/μl (range 3.98-12.98 x105/μl) and mean leukocyte count was 3.34±1.12 x103/ μl in PRP. Average consumption of analgesic (Tramadol) was much higher in saline group (21.33±2.79 days) as compared to PRP groups (5.39 ±1.45 days) (P=0.001).

## Discussion

The main takeaways from this study were that PRP injections given intra-articularly yielded significant improvement in pain and functional parameters (VAS and WOMAC) sustaining for a period of 9 months in KOA grade II,III patients in comparison to placebo (NS), regardless of number of injections, which reinforces the evidence favouring the efficacy of PRP as an effective treatment for KOA. Our findings are consistent with several previous studies^9-13^ and supported by recent meta-analyses and systematic reviews^14-16^ all of which have reported that the patients treated with PRP injections exhibited better clinical outcomes compared to those treated with control, hyaluronic acid (HA), or steroids, in short term and long term (1–2 years). In a recent multi centre RCT by Chu *et al*^17^, more than 600 participants with KOA were given three PRP injections three weeks apart and they found it to be very effective in improving clinical scores (WOMAC pain and function score, VAS) over 60 months. They also observed lower levels of synovial TNF-α and IL-1β along with reduced deterioration of knee joint cartilage volume in PRP group compared to placebo group. In another RCT comparing the results of plasma rich in growth factors (PRGF) and PRP with ozone and HA, PRGF and PRP exhibited better therapeutic results at one year^18^. Although we have not assessed the radiological outcome, but with respect to clinical outcomes, our findings concur with above studies, showing PRP to be very effective in providing significant pain relief and functional improvements over placebo, with both the evaluated outcome scores reaching clinically significant levels (MCIC of >12% of baseline and ≥2cm difference of mean for WOMAC and VAS, respectively) at every follow-up (3, 6 and 9 months post intervention) in grade II and III KOA. The exact cause of OA is still unclear but the major mechanism of joint damage and pain is understood to be up-regulation of catabolic and down-regulation of anabolic processes leading to a cascade of events where in pro inflammatory mediators like interleukins (IL) (IL-1β, IL-6, IL-15, IL-17, IL-18), tumour necrosis factor (TNF)-α, leukaemia inhibitory factor (LIF), matrix metalloproteinases (MMPs), reactive oxygen species (ROS) and prostaglandins are increased, which not only mediate joint degradation but also result in inadequate synthesis of proteoglycans, collagen, anti-inflammatory cytokines (IL-10, IL-4), and growth factors^3,4^. In addition, other Inflammatory mediators, such as bradykinin, histamine, substance P, have also been implicated, which induce hyperalgesia by a number of direct and indirect actions^20,21^. The pain relief in OA after PRP injection is achieved because the growth factors and other biomolecules in PRP not only have anti-inflammatory but also have anti-nociceptive properties (via inhibition of intra-cellular signalling NF-KB)^7,22,23^. In addition, PRP may also lead to a change in macrophages from inflammatory (M1) to reparative (M2) phenotype, resulting in reduction of inflammation and stimulation of tissue repair^24^. PRP is also believed to activate the anti-oxidant response thereby preventing further damage by ROS, through the intra-cellular signalling pathway Nuclear factor erythroid two related factor 2-antioxidant response element (NrF2-ARE) in osteoblasts^25^. Apart from its anti-inflammatory properties, PRP also has been shown to possess biolubricating effect by stimulating chondrocytes and synoviocytes into producing HA, as well as through the superficial zone protein (SZP) or lubricin^26,27^.

Coll2-1, a degradation product of collagen type 2 (predominant component of cartilage ECM), is thought to be a specific marker for hyaline cartilage^28^. Coll2-1 amounts have been found to be increased in OA due to breakdown of cartilage, mediated by catabolic processes and hence it has the potential to be used not only as disease progression marker^28,29^, but also as a predictor for treatment response^28,30^. Coll2-1 is also thought to be an active contributor to the disease process and has been identified as a therapeutic target^31^. Our analysis of serum Coll2-1 levels at baseline and nine months post intervention showed a significant reduction in its levels in all three PRP groups while as the change in saline group was found to be non-significant. Three doses seem to produce better reduction in serum levels of Coll2-1 than one or two doses. This indicates PRP may have some chondroprotective effect which can be accentuated by multiple doses. These findings are similar to those reported by Fawyzy *et al*^30^, who observed a significant decrease in serum Coll2-1 concentrations over a three month period with a single dose of PRP, but this study was limited by the lack of a control group and a short follow-up. In another randomised study^32^, significant reduction in serum Coll2-1 were achieved with multiple serial injections of growth factor concentrate (GFC), a form of activated PRP, compared to placebo in KOA grade II and III. In light of these findings, reduced levels of Coll2-1 post treatment may be indicative of improvement in overall joint microenvironment and hence may relate to the clinical improvement as well. However, it is difficult to assess exactly as to how reduction in serum Coll2-1 levels may translate to improvement of clinical outcomes. Although Coll2-1 can be released from any arthritic joint but Deberg *et al*^33^ reported serum Coll2-1 concentrations decreased three months after knee arthroplasty, indicating its sensitivity to structural changes in a single joint. Moreover, we have included patients presenting with pain in the knee joint only. Platelets contain a plethora of growth factors and among them TGF-1β and IGF-1 mainly are thought to be involved in synthesis of type II collagen and proteoglycans and they also induce the chondrogenic differentiation of MSCs^7,8^. PRP lead to a significant reduction of serum Coll2-1 levels at final follow-up in our study and these effects were seen irrespective of frequency (single, double or triple dose). On inter PRP group comparison, multiple doses (two or three) spaced over one month produced significantly better clinical results than a single dose but the decrease in Coll2-1 was significant only for those who received three injections vs those who received a single injection. The reduced levels of Coll2-1 post intervention with PRP thus may indicate that it may have some chondroprotective effect, although it has to be supplemented by radiological evidence as well. Despite all this, its important to acknowledge that the use of PRP in KOA still remains a topic of discussion and debate particularly with respect to its chondrogenic potential. In a placebo controlled trial of 288 patients with mild to moderate KOA, Bennell *et al*^34^ concluded serial PRP injections do not lead to any significant change in knee joint cartilage thickness nor in clinical outcomes compared to placebo over a period of one year. Prodromidis *et al*^35^ reported the evidence currently is not strong enough to use PRP as a chondrogenic agent for KOA.

The heterogenicity in the studies are mainly because of lack of standardisation of PRP with respect to the number, frequency, type (LP, LR, activated, inactivated), concentration of platelets and growth factor amount. Through current study, we have attempted to compare and assess the results of single dose vs multiple doses (two and three injections spaced over one month) of PRP injections. Yurtbay *et al*^36^ assessed the effects of single and three doses of PRP at monthly intervals and reported significantly better clinical scores with PRP than placebo at three-months, six-months and one year with no difference across groups at two years and triple injections were found to perform better than one dose. Tavassoli *et al*^19^ found two injections of PRP (given at three-week intervals) resulted in superior outcomes of VAS and WOMAC over single PRP and 3 HA doses, however no blinding of participants was done in this study, they were followed-up for 12 weeks only and no control group was used. Gormeli *et al*^37^ reported similar results, with three doses of PRP performing better than a single dose of HA, PRP or saline for patients with early KOA. This was echoed in a recent meta-analysis by McLarnon *et al*^14^, where three IA PRP injections (weekly) were found to be superior than one injection. The results from another study have found that although four PRP injections given six weeks apart did not lead to any significant difference of synovial cytokines or growth factors compared to two injections, improved clinical outcomes were seen in both the groups for periods of up to one year, but this study lacked a control group nor it was randomised^38^. According to a meta-analysis by Vilchez-Cavazos *et al*^39^, single PRP injections were as effective as multiple (two or three) for pain improvement but multiple seemed to provide better joint functionality at six months. The findings in our study conform to the above studies with multiple (two or three doses) PRP injections given at one monthly intervals proving superior to a single injection in ameliorating patient reported clinical scores of pain and function at three, six, and nine months.

Kon *et al*^40^ and Filardo *et al*^41^ have reported younger patients to perform better while as others have stated no effect of age on the outcome. Yurtbay *et al*^36^, found patients aged 51–60 years performed better than older individuals, whereas those aged below 55 performed better in our study. This may be because of higher levels of GFs as well as lower baseline scores in this group. The effect of sex and BMI on outcome is variable with studies finding no differences between sexes despite having a greater percentage of female patients^10,11,13^. Patel *et al*^11^ reported no effect of BMI on outcome while as others^12^ have reported better results in patients with low BMI. Despite having predominantly female and overweight population in our study, we did not find any difference in outcome scores among them, however, it cannot be ascertained as the patient distribution was not homogeneous in these groups.

Previous evidence indicates the adverse effects with the use of IA PRP tend to be mild and self-limiting in the form of pain, synovitis and erythema, with no reports of serious complications^14^. Apart from mild synovitis in three patients with first injection which resolved with conservative measures in few days, no other adverse events were seen.

This study had some limitations in the form of a small sample size of each group but the study was adequately powered as sample size calculation was done before recruitment. The outcome scores were subjective rather than being objective and the follow-up was short (nine months). Radiography was done for diagnosis and grading at the start and no radiological assessment was done with MRI to look for articular cartilage changes. We also did not perform the biochemical analysis for GF estimation and look for changes in joint biology. Future high quality RCTs with objective outcome measures in conjunction with radiological assessment and biochemical analysis with highly sensitive and specific biomarkers are needed to further evaluate the efficacy of PRP and dosing in treatment of OA knee.

## Conclusions

Autologous PRP injected intra-articularly, whether given as a single dose or multiple doses, results in safe and clinically significant amelioration of subjective clinical outcomes of pain and function in K-L grade II,III KOA with benefits lasting for at least nine months. PRP also lead to a significant decrease in serum concentration of cartilage degradation biomarker Coll2-1 and these benefits can be accentuated by multiple doses (three injections resulting in more pronounced decrease than a single injection) spaced at monthly intervals.
